# Targeting S100B in Cerebral Ischemia and in Alzheimer's Disease

**DOI:** 10.1155/2010/687067

**Published:** 2010-09-02

**Authors:** Takashi Mori, Takao Asano, Terrence Town

**Affiliations:** ^1^Department of Biomedical Sciences, Saitama Medical Center and University, 1981 Kamoda, Kawagoe, Saitama 350-8550, Japan; ^2^Department of Pathology, Saitama Medical Center and University, 1981 Kamoda, Kawagoe, Saitama 350-8550, Japan; ^3^Department of Biomedical Sciences, Regenerative Medicine Institute, Cedars-Sinai Medical Center, 8700 Beverly Boulevard, Los Angeles, CA 90048, USA; ^4^Department of Neurosurgery, Maxine Dunitz Neurosurgical Institute, Cedars-Sinai Medical Center, 8700 Beverly Boulevard, Los Angeles, CA 90048, USA; ^5^Department of Medicine, David Geffen School of Medicine, University of California, Los Angeles, 8700 Beverly Boulevard, Los Angeles, CA 90048, USA

## Abstract

S100B is an EF-hand calcium-binding protein that exerts both intracellular and extracellular effects on a variety of cellular processes. The protein is predominantly expressed in the central nervous system by astrocytes, both physiologically and during the course of neurological disease. In the healthy adult brain and during development, constitutive S100B expression acts as a trophic factor to drive neurite extension and to referee neuroplasticity. Yet, when induced during central nervous system disease, the protein can take on maladaptive roles and thereby exacerbate brain pathology. Based on genetic and pharmacological lines of evidence, we consider such deleterious roles of S100B in two common brain pathologies: ischemic stroke and Alzheimer's disease (AD). In rodent models of ischemic brain damage, S100B is induced early on during the subacute phase, where it exacerbates gliosis and delayed infarct expansion and thereby worsens functional recovery. In mouse models of AD, S100B drives brain inflammation and gliosis that accelerate cerebral amyloidosis. Pharmacological inhibition of S100B synthesis mitigates hallmark pathologies of both brain diseases, opening the door for translational approaches to treat these devastating neurological disorders.

## 1. Introduction

The principal cell types comprising the brain parenchyma are neurons and glial cells. The term “glia” is customarily used to refer to neuroglia (comprised of astrocytes, oligodendrocytes, and more recently, NG2 oligodendrocyte progenitors), Schwann cells, and central nervous system- (CNS-) resident macrophages known as microglia. Occasionally, ependymal cells (ependymoglia) are also classified as glia, as they are differentiated from radial glia [1] and share astrocytic properties [2]. In addition to parenchymal cells, cerebral vascular cells exist and form a physiological barrier in the CNS known as the blood-brain barrier (BBB). Among these cellular constituents, astrocytes greatly outnumber neurons in the brain, making up about 50% of human brain volume [3]. Despite the time-honored concept that astrocytes are “silent partners of the working brain”, accumulating evidence has shown that astrocytes are active participants in CNS physiology [4–6], including transport of substances between blood and neurons [3, 4], cerebral blood flow metabolism control [7–10], modulation of synaptic transmission [11–13], synaptogenesis [14–18], and neurogenesis [19–22].

Yet, astrocytes are capable of directly endangering neurons during the course of inflammatory CNS disorders [23, 24]. In fact, acute and chronic CNS disorders often have a component of glial activation, characterized by infiltration of activated microglia and astrocytes into the region of damaged tissue [21, 25–28]. Reactive astrocytes likely exert their effects in collaboration with activated microglia. On the one hand, these cells may exacerbate neuroinflammation by producing a myriad of toxic substances, including cytokines, nitric oxide, prostanoids, and reactive oxygen species; on the other hand, they are capable of exerting beneficial effects by producing neurotrophic substances [3–6, 21, 25, 29, 30]. Much recent attention has been focused on this enigmatic duality so often observed in studies of activated glia within the broader context of neurological and neurodegenerative diseases.

This paper begins by addressing the double-edged sword of both beneficial and detrimental actions of astrocytic S100B in the CNS. Subsequently, we move on to focus on contributions of reactive astrocytes to glial inflammatory responses in two common neurodegenerative diseases: cerebral ischemia and Alzheimer's disease (AD). Finally, we consider the concept of translating S100B inhibition to the clinic for the treatment of neurodegenerative diseases.

## 2. Beneficial and Detrimental Actions of S100B in the Central Nervous System

S100 is a large family (over 20 members) of EF-hand (helix E-loop-helix F) calcium-binding proteins, and all but four are clustered on human chromosome 1q21, while the human gene encoding S100B maps to chromosome 21q22 [31–38]. A total of ten S100 family members are expressed in the brain, including S100A1, S100A2, S100A4, S100A5, S100A6, S100A10, S100A11, S100A13, S100B, and S100Z. In addition, mRNA levels of S100A1/S100B are 5-fold higher than S100A6/S100A10 and 100-fold higher than S100A4/S100A13 in the mouse brain. Five of these six family members (S100A1, S100A6, S100A10, S100A13, and S100B) are increased in an age-dependent manner in adult mice [39]. S100B is detected in varying abundance in a limited number of brain cells including astrocytes, maturing oligodendrocytes, neuronal progenitor cells, pituicytes, ependymocytes, and certain neural populations. Although the majority of astrocytic S100B localizes within the cytoplasm, 5%–7% is membrane bound [32, 34, 38, 40–42]. S100B has been implicated in Ca^2+^-dependent regulation of a variety of intracellular functions such as protein phosphorylation, enzymatic activity, cell proliferation and differentiation, cytoskeletal dynamics, transcription, structural organization of membranes, intracellular Ca^2+^ homeostasis, inflammation, and protection against oxidative damage [31–38, 43–46].

Binding of S100B to receptors on target cells releases intracellular free Ca^2+^ from Ca^2+^ stores via activation of phospholipase C and downstream inositol triphosphate [43]. As overexpression of S100B induces downregulation of p53 protein [47], calcium signaling and S100B may act in cooperation with this pathway, which is implicated in growth inhibition and apoptosis [47–49]. Yet, how elevation of cytosolic Ca^2+^ transduces S100B binding into trophic and proliferative effects on brain cells is still elusive.

During brain development, a temporal correlation has been reported between synaptogenesis and astrocyte differentiation [50]. Numerous findings support the notion that astrocytes regulate the formation, maturation, and maintenance of synapses [14–17]. Astrocytic S100B expression increases in the rodent brain during the first 3 postnatal weeks—a critical period for glial proliferation and synaptogenesis, and it was suggested nearly 40 years ago that the protein likely referees synaptic development *in vivo* [51, 52]. In the adult rodent brain, S100B expression persists at nanomolar concentrations and likely orchestrates neurite extension [53], enhances survival of neurons and promotes synapse formation [54], and exerts protective actions after injury [55, 56].

On the other hand, there is accumulating evidence that S100B may also have detrimental actions in the CNS. Activation of microglia triggers and promotes astrocytic activation through release of cytokines such as tumor necrosis factor-*α* (TNF-*α*) and interleukin-1*β* (IL-1*β*). These and other cytokines drive a synergistic relationship between these two types of glial cells via a vicious positive feedback loop known as the “cytokine cycle” [24]. While numerous bioactive substances are part and parcel of this cycle, S100B can be regarded as a major constituent of this brain-damaging feedback loop. In this context, Hu and colleagues demonstrated that S100B at micromolar concentrations induces neuronal damage in a neuron and astrocyte coculture experiment by causing overexpression of inducible nitric oxide synthase and subsequent release of nitric oxide [57]. Lam and colleagues showed that S100B stimulates inducible nitric oxide synthase in rat primary cortical astrocytes through a signal transduction pathway that involves activation of the transcription factor nuclear factor-*κ*B (NF-*κ*B), which is a master regulator of pro-inflammatory responses [58]. Hu and Van Eldik showed that S100B upregulates IL-1*β* expression in astrocytes [59], and Ponath and coworkers showed that S100B stimulates release of IL-6 and TNF-*α* from astrocytes [60].

Importantly, numerous secreted S100 proteins (S100B, S100A1, S100A2, S100A4, S100A5, S100A6, S100A7, S100A8/A9, S100A11, S100A12, and S100P) can act in either an autocrine or paracrine fashion through a common receptor: the receptor for advanced glycation endproducts (RAGE), a multiligand receptor that belongs to the immunoglobulin family. Moreover, in cell-based assays, all these family members (except for S100A2 and S100A5) have been shown to trigger RAGE-dependent signaling [38, 61–65]. The fact that the RAGE promoter has functional NF-*κ*B binding sites reinforces the likelihood that this signaling pathway is an important trigger of inflammatory pathogenesis [66]. Moreover, it has been shown that RAGE and S100/calgranulin signaling propagate, recruit, and activate cellular pro-inflammatory effectors [61]. Accordingly, nanomolar concentrations of extracellular S100B can trigger expression of the anti-apoptotic factor Bcl-2 in RAGE-expressing cells, whereas micromolar S100B concentrations induce apoptosis via RAGE activation [67]. In primary microglia, S100B stimulates IL-1*β* production by activating extracellular signal-regulated kinase 1/2 (ERK1/2), p38 mitogen-activated protein kinase (p38 MAPK), and c-Jun NH_2_ terminal protein kinase (JNK) [68]. There is evidence that S100B-mediated microglial pro-inflammatory responses are RAGE-dependent, as RAGE ligation by S100B induces expression of the pro-inflammatory enzyme cyclooxygenase-2 (COX-2) via parallel Ras-Cdc42-Rac1-dependent activation of JNK and Ras-Rac1-dependent stimulation of NF-*κ*B transcriptional activity. Further, S100B engagement of RAGE coordinately stimulates NF-*κ*B and AP-1 transcriptional activity and synergizes with IL-1*β* and TNF-*α* to upregulate COX-2 expression [65].

## 3. Reactive Astrocytes and S100B in Cerebral Ischemia

While there is a wide spectrum of various forms of brain damage, ischemic stroke (cerebral ischemia) is the most prevalent type of brain injury that causes death and long-lasting disability. The pathogenic effects of microcirculatory dysfunction in cerebral ischemia can be divided into two categories: impairment of intraluminal cerebral blood flow and extraluminal effects resulting from alterations in the BBB [69]. Over the past decade, extensive research efforts have been directed toward development of neuroprotective drugs against cerebral ischemia, such as ion channel antagonists, glutamate receptor antagonists, and free radical scavengers. Anti-platelet drugs, anti-thrombotic drugs, tissue plasminogen activators, and free radical scavengers have already come into routine clinical use and clinical trials investigating additional agents are ongoing.

In the clinical setting, S100B is a well-known biomarker that positively associates with severity of brain damage and has been shown to predict prognosis after subarachnoid hemorrhage [70, 71], ischemic brain injury [72, 73], and traumatic brain injury [74]. It is important to note that the above clinical studies are correlative in nature and presume that rise in cerebral spinal fluid and serum concentrations of S100B is due to release of intracellular stores of the protein upon cellular disruption. Thus, the possibility that S100B is synthesized *de novo* by reactive astrocytes and exacerbates infarct evolution was not considered in these reports. In addition, a surge of attention from both physiological and therapeutic standpoints has been directed toward the possible roles of astrocytes in neurometabolic and neurovascular coupling.

**Figure 1:**
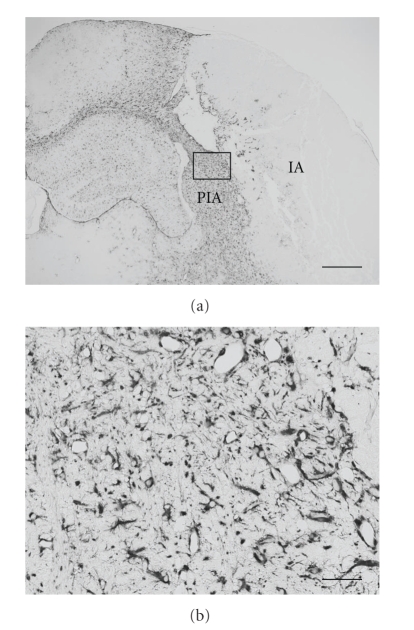
Reactive astrocytes, earmarked by enhanced expression of glial fibrillary acidic protein, hyperplasia, and gemistocytic changes, form a glial barrier in the peri-infarct area of a mouse brain at 7 days post-focal cerebral ischemia. Higher magnification image (b) represents the inset of the lower magnification coronal brain section (a). Abbreviations used: IA: infarct area; PIA: peri-infarct area. Bars denote 500 *μ*m (a) and 50 *μ*m (b).

After focal cerebral ischemia, brain damage is accompanied by infiltration of reactive astrocytes into the peri-infarct area. Specifically, astrocytes are copiously activated along the outer border of the infarct. Reactive astrocytes are earmarked by increased expression of S100B and glial fibrillary acidic protein (GFAP), appear around 24 hours after the onset of ischemia, and undergo hypertrophy and hyperplasia for a period of weeks thereafter [75–77]. During the chronic disease phase (>168 hours post-ischemia), astrocytes extensively wall off the BBB at the glial limitans [78] (Figure 1), participate in angiogenesis concurrent with development of cerebral edema [29, 79], and possibly drive neurogenesis [19, 22]. On the other hand, when considering the subacute phase (24–168 hours after ischemia onset) of this neurological disorder, it has been difficult to assign a role to reactive astrocytes. This is owed to the long-held belief that infarct expansion after focal cerebral ischemia comes to a complete halt by 12 hours, well before astrocytic activation manifests [80]. However, “delayed infarct expansion” during the subacute phase of cerebral ischemia was later discovered and is now becoming generally accepted. This pathological event has led to a wider recognition of the possible detrimental role of astrocytic activation in the subacute phase of cerebral infarction as a valid research target [81].

With regard to the concept of delayed infarct expansion, Garcia and colleagues used the rat permanent focal cerebral ischemia model and noted for the first time in 1993 that there was a significant increase in infarct area during the time interval between 6 and 72 hours after ischemia onset [76]. In 1996, Du and coworkers followed up by showing that infarction after mild transient focal cerebral ischemia could develop in a delayed fashion in the rat [82]. Using the rat permanent focal cerebral ischemia model, we have shown that after the rapid expansion phase during the first 24 hours post-insult, infarct volume continues to slowly but steadily increase until it reaches a peak at 168 hours. At this time-point, there is a significant increase in infarct volume of ~41% as compared to 24 hours post-insult. Moreover, the occurrence of delayed infarct expansion is associated with astrocytic activation as well as increased abundance of S100B in the peri-infarct area [81]. Clinical research relying on magnetic resonance imaging of ischemic stroke patients has disclosed that infarct expansion occurs during two distinct phases (acute and delayed) [83–85], and the time course and magnitude of ischemic enlargement are quite comparable between patients and the rat permanent focal cerebral ischemia model [81]. The above lines of evidence reinforce the idea that delayed infarct expansion is a viable therapeutic target and may even be as important as acute infarct expansion in terms of disease progression. Moreover, the association among the appearance of numerous peri-infarct reactive astrocytes, increased abundance of S100B in the peri-infarct area, and occurrence of delayed infarct expansion has emerged as an issue of high clinical importance.

Based on the work discussed above, we undertook a series of experiments to investigate the putative causal relationship between S100B and exacerbation of brain damage. Specifically, we forced expression of human S100B or pharmacologically blocked S100B biosynthesis in rodents subjected to ischemic brain injury. We discuss our relevant findings to follow.

### 3.1. Human S100B Exacerbates Ischemic Brain Damage and Peri-Infarct Gliosis

We sought to evaluate whether forced expression of human S100B in astrocytes may exacerbate brain damage and delayed infarct expansion after permanent focal cerebral ischemia. As a corollary, we aimed to examine whether severity of delayed infarct expansion and peri-infarct gliosis were correlated. To experimentally probe these questions, we utilized transgenic mice overexpressing human S100B (Tg huS100B mice; carrying approximately 10 copies of the human S100B gene under endogenous regulatory control) on an outbred CD-1 genetic background. These transgenic mice overexpress S100B in cortical astrocytes by 4-6-fold over endogenous wild-type levels [86]. We permanently induced focal cerebral ischemia by tandem occlusion of the left common carotid artery and distal segment of the middle cerebral artery using an electrocoagulation method under normothermia [87]. Notable results of this experiment are that Tg huS100B versus control CD-1 mice show significant exacerbation of infarct volume, neurological deficits, and peri-infarct-reactive gliosis (astrocytosis and microgliois) during the time interval between 1 and 7 days after the onset of ischemia, providing evidence for increased susceptibility of Tg huS100B mice to ischemic brain stress. Moreover, reactive gliosis as indicated by increased S100, GFAP, and Iba1 immunoreactivity in the peri-infarct area continued to increase in Tg huS100B mice through to 7 days after focal cerebral ischemia, whereas control mice reach a plateau at 3 days following ischemic insult.

Our interpretation of the above evidence is that pro-inflammatory events associated with S100B-accelerated glial activation in the peri-infarct area contribute to exacerbation of brain damage. In further support of this conclusion, we noted a positive correlation between reactive gliosis along the infarct border and occurrence of delayed infarct expansion in Tg huS100B mice, but no significant relationship between these variables was detected in control animals. Together, these results support the notion that enhanced and prolonged activation of glial cells plays a detrimental role during the subacute phase (1 to 7 days) of focal cerebral ischemia in the rodent brain [81, 87, 88]. Moreover, our results also bolster the hypothesis that astrocyte-derived S100B is a pivotal mediator of these deleterious effects [88–90]. In addition, we found that Tg huS100B mice had higher constitutive levels of S100 as compared with CD-1 mice. The distinction between constitutive and ischemic brain injury-enhanced S100 is important. This is because increased baseline S100 abundance may predispose to worse acute infarct expansion within 1 day after ischemic induction, whereas further induction of S100 expression likely promotes exacerbation of delayed infarct expansion.

As mentioned above, astrocytes and astrocyte-derived S100B seem to play dichotomous roles in progression of various CNS pathologies, although it is still controversial whether, on average, detrimental effects outweigh neuroprotective effects or *vice versa*. Since S100B can be trophic to glia [91], this effect may mechanistically underlie hyperplasia of peri-infarct reactive glia after focal cerebral ischemia in Tg huS100B mouse brains. Interestingly, aged rats show accelerated glial reactivity after cerebral ischemia, which coincides with impaired functional recovery [92]. Thus, worsening of neurological deficit in Tg huS100B mice after focal cerebral ischemia may be caused by inappropriately accelerated “trophic” glial responses.

The above studies adopted a genetic approach to demonstrate *in vivo* evidence that forced expression of human S100B exacerbates brain damage, neurological deficits, and peri-infarct reactive gliosis after ischemic stress. However, whether S100B sits at the epicenter of the damaging cytokine cycle and delayed infarct expansion needed further verification. To definitively validate this hypothesis, we examined whether pharmacological blockade of S100B biosynthesis would produce converse effects on ischemic brain damage. As detailed below, we found support that pharmacological strategies aimed at inhibiting S100B abundance would, in principle, be beneficial to mitigate cerebral ischemia.

### 3.2. Suppressing S100B Synthesis Mitigates Cerebral Ischemic Brain Damage

To examine if enhanced synthesis of S100B by peri-infarct reactive astroglia played a role in delayed infarct expansion, we undertook a pharmacological approach using a novel agent, arundic acid [ONO-2506, (*R*)-(–)-2-propyloctanoic acid, ONO Pharmaceutical Co. Ltd.], which has been shown to suppress astrocytic S100B synthesis [93]. Yet, arundic acid exerts additional effects both *in vitro* and *in vivo* on other biomolecules, including inhibition of nerve growth factor-*β*, inducible nitric oxide synthase, and COX-2 expression and increasing glutathione synthesis, mRNA expression of glutamate transporters (glutamate transporter subtype 1: GLT-1 and glutamate/aspartate transporter: GLAST) and GABA receptors (GABA_A_-R *β*1, GABA_A_-R *β*2, and GABA_A_-R *β*3) in activated astrocytes. Yet, it is still unclear whether the agent modulates these other proteins directly or indirectly via its suppressive effect on S100B [93]. To model ischemic brain injury in the rat, we permanently occluded the left middle cerebral artery under normothermia and then divided at a site proximal to the origin of the lenticulostriate arteries [81]. Intravenous administration of arundic acid (10 mg/kg, once a day), initiated immediately after induction of ischemia, significantly reduced infarct volume at 168 hours (but not at 72 hours) in this rat focal cerebral ischemia model. Surprisingly, the agent did not inhibit acute infarct expansion during the initial 24 hours, but almost completely inhibited delayed infarct expansion.

In a subsequent experiment designed to elucidate a therapeutic window, rats were allocated to groups that received the first administration of drug at 24, 48, or 72 hours after focal cerebral ischemia, and infarct volumes were compared at 168 hours. Treatment with arundic acid commenced at 24 or 48 hours after the induction of ischemia significantly decreased infarct volumes at 168 hours by approximately 43% and 35%, respectively. However, when treatment was initiated later on at 72 hours, we did not observe a protective effect. Collectively, these results show an encouraging liberal therapeutic window, which coincides with the peak of astrocytic S100B synthesis induction after focal cerebral ischemia. Numbers of apoptotic cells in the ischemic hemisphere at 72 hours post-focal cerebral ischemia were markedly decreased by arundic acid treatment. In addition, tissue levels of S100B as well as GFAP in the peri-infarct area were significantly decreased by the agent. Thus, administration of arundic acid led to significant attenuation of astrocytic S100B synthesis, general inhibition of astrocytic activation, and reduced numbers of apoptotic cells in the peri-infarct area. Notably, delayed infarct expansion was almost completely inhibited in this experimental disease model. In addition, we found significant improvement in neurological scores and spontaneous activities as early as one day after the first drug treatment, and these beneficial outcomes continued for several days after treatment. These results support the concept that pharmacological inhibition of astrocytic S100B limits occurrence of delayed infarct expansion after focal cerebral ischemia.

As arundic acid does not significantly inhibit infarct expansion in the rat focal cerebral ischemia model when given beyond the 72-hour time-point, the above results indicate that the compound acts to improve later brain dysfunction through a mechanism that is temporally unrelated to inhibition of subacute infarct expansion. In this regard, it has been reported that transient focal cerebral ischemia is accompanied by relatively widespread and persistent functional disturbances in the peri-infarct region [94, 95]. Thus, improvement in neurological deficit observed after the subacute phase is likely ascribable to the beneficial actions of arundic acid on functional disturbances in the ischemic hemisphere as well as on neural plasticity in both ischemic and non-ischemic hemispheres. That neural functional derangement and delayed infarct expansion can be simultaneously mitigated by arundic acid raises the possibility that both pathologies stem from a common pathogenic mechanism: astrocytic activation. With regard to the relationship between these pathologies, it has been reported that symptoms can regress despite increase in infarct volume [25]. Results of this experiment extend the notion that pharmacological modulation of astrocytic activation via inhibiting S100B biosynthesis can have long-lasting effects on functional recovery after injury, despite only reducing infarct expansion during the initial subacute phase.

**Figure 2:**
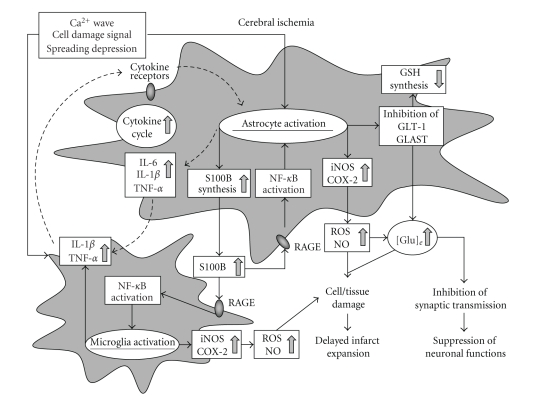
A model for the roles of activated astrocytes, activated microglia, and S100B in delayed infarct expansion after focal cerebral ischemia. The glial cytokine cycle is represented by *dotted lines*. Abbreviations used: COX-2: cyclooxygenase-2; [Glu]_e_: extracellular glutamate; GLAST: glutamate/aspartate transporter; GLT-1: glutamate transporter subtype 1; GSH: glutathione; IL-1*β*: interleukin-1*β*; IL-6: interleukin-6; iNOS: inducible nitric oxide synthase; NF-*κ*B: nuclear factor-*κ*B; NO: nitric oxide; TNF-*α*: tumor necrosis factor-*α*; RAGE: receptor for advanced glycation endproducts; ROS: reactive oxygen species.

The above lines of evidence from (1) a genetic model of forced human S100B expression and (2) pharmacological blockade of S100B biosynthesis bolster a causal relationship between S100B and exacerbation of ischemic brain damage. After cerebral ischemia, astrocytes change in shape and function within a relatively short time-frame, and likely exert their effects at multiple levels and at different phases of infarct evolution. During the subacute phase of focal cerebral ischemia, reactive astrocytes seem to promote delayed infarct expansion by enhancing the viscous cytokine cycle in collaboration with activated microglia. The work detailed above reinforces the idea that astrocytic synthesis of S100B plays a central role in the pathobiology of this disease, and prompts a mechanistic model wherein activated astrocytes, activated microglia, and S100B participate in brain damage and delayed infarct expansion after focal cerebral ischemia (Figure 2). A similar pathogenic mechanism seems to exist during the course of chronic neurodegeneration after focal cerebral ischemia in remote areas such as the thalamus, striatum, and substantia nigra. Since augmented astrocytic S100B synthesis accompanies a diverse number of brain pathologies, we moved on to consider the role(s) of this enigmatic molecule in neurodegenerative disease. In particular, we focused on of the relationship between S100B and AD pathology, as detailed to follow.

## 4. Alzheimer's Disease, Neuroinflammation, and S100B

AD is the most common progressive dementia of aging and is characterized by memory loss and gradual decline in cognition. AD neuropathological hallmarks include brain deposition of amyloid-*β* (A*β*) peptide as senile plaques, accumulation of abnormal *tau* protein filaments as intracellular neurofibrillary tangles, extensive neuronal degeneration and loss, profound synaptic loss, and *β*-amyloid plaque-associated astrocytosis and microgliosis [96–98]. Moreover, augmented expression of S100B has been reported in the brains of patients with Down's syndrome and in AD [99]. In AD, it has been shown that S100B abundance is elevated in activated astrocytes colocalized with *β*-amyloid plaques, where progressive expression of IL-1*α* by activated microglia has also been noted [100]. Importantly, it has been reported that astroglial overexpression of S100B actually precedes appearance of neuritic *β*-amyloid plaques in the PDAPP mouse model of AD [101], suggesting that S100B overabundance may drive cerebral *β*-amyloidosis as opposed to existing as an epiphenomenon.

Brain A*β* deposition “normally” occurs during the course of senile changes [102], and it is probably a combination of increased accumulation/reduced clearance of the peptide and impaired ability to cope with toxic downstream effects of A*β* that drive AD. A*β* is produced from sequential endoproteolytic cleavage of the type 1 transmembrane glycoprotein, *β*-amyloid precursor protein (APP), by *β*- and *γ*-secretases [103–106]. Recently, it has been hypothesized that newly produced A*β* enters a dynamic equilibrium between soluble and deposited forms in the brain, with continual transport of soluble A*β* out of the brain and into the circulation [107]. In fact, cerebral amyloidosis in AD patient brains might be licensed by an imbalance in this dynamic equilibrium.

Rooted in the “amyloid cascade hypothesis” of AD, which purports that accumulation of cerebral A*β* sets into motion a series of toxic downstream events [108, 109], extensive research efforts have been directed toward development of anti-amyloid therapies aimed at reducing cerebral A*β* production (e.g., *β*- or *γ*-secretase inhibitors) [110–113] or enhancing A*β* clearance by targeting brain immune/inflammatory mechanisms [27, 28, 114–123]. Unfortunately, however, agents that demonstrated benefit in rodent models of AD have not yet lived up to their promise in the clinic, where cholinesterase inhibitors (e.g., donepezil, rivastigmine, tacrine, and galantamine) and N-methyl D-aspartate antagonist (e.g., memantine) continue to be prescribed. It should be noted, however, that these currently indicated drugs produce only modest symptomatic benefit, especially when administered in the advanced stage of the disease. Thus, research into new drugs based on alternative therapeutic targets has continued at a feverish pace.

Over the past decade, we and others have proposed that inflammatory and immune response pathways are chronically activated in AD patient brains at low levels, and likely play a role in disease progression. “Inflammation” is canonically defined as edema and tissue infiltration of neutrophils, lymphocytes, plasma cells, and macrophages, but these classical pathological findings are not present in the post-mortem AD brain. Yet, accumulating evidence indicates that a variety of factors known to be major participants in inflammatory and immune responses are the norm in AD. Chronic activation of glial cells in and around *β*-amyloid plaques may be pathoetiologic in AD via production of numerous neurotoxic acute-phase reactants, pro-inflammatory cytokines, and immunostimulatory molecules [24, 28, 124, 125]. However, despite low-level, chronic activation of innate immunity and inflammatory responses in the AD brain, glial cells ultimately fail to clear cerebral *β*-amyloid plaques.

**Figure 3:**
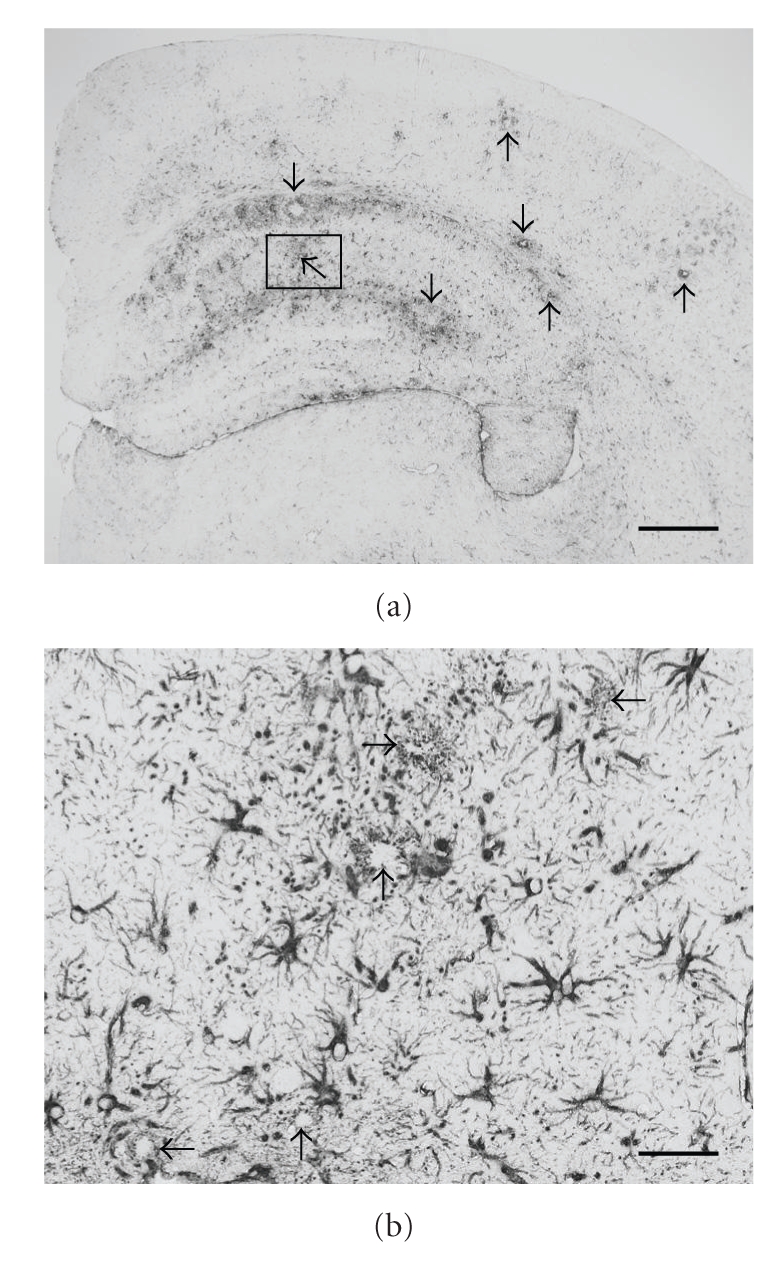
Numerous *β*-amyloid plaque-associated reactive astrocytes (positive for glial fibrillary acidic protein) are shown in a coronal brain section from a Tg2576 Alzheimer's disease model mouse at 19 months of age. Higher magnification image (b) represents the inset of the lower magnification coronal brain section (a). Arrows show *β*-amyloid plaques that are surrounded by glial fibrillary acidic protein-positive processes in the plaque periphery. Bars denote 500 *μ*m (a) and 50 *μ*m (b).

Astrocytes and microglia are the main innate immune response effectors in the CNS. Imbalances between protective and destructive functions of these cells might impact neurotoxicity and/or synaptotoxicity in the context of neurodegenerative disease. In the AD brain, reactive astrocytes and microglia co-exist in both temporal and spatial proximity with *β*-amyloid plaques (Figure 3), and it is thought that diffuse *β*-amyloid deposits attract and activate IL-1*β*-secreting microglia, which in turn activate astrocytes and promote astrocyte-derived S100B synthesis. This self-propagating neuroinflammatory loop promotes further release of pro-inflammatory cytokines and acute-phase reactants by both activated microglia and astrocytes, leading to production of oxyradicals and nitric oxide, which are toxic at supraphysiologic levels. Aside from further enhancing inflammatory responses, these pro-inflammatory substances likely induce bystander neuronal injury in the AD brain [24, 124].

Given the conspicuous role of brain inflammation and activated astrocytes in AD pathobiology, we undertook genetic and pharmacological approaches to target S100B in order to explore a putative causal relationship between S100B and progression of AD-like pathology. Our relevant findings are reviewed and discussed in the following sections.

### 4.1. Overexpression of Human S100B Exacerbates Alzheimer's Disease-Like Pathology

Over the past decade, numerous transgenic mouse models have been constructed using mutations in human APP and/or presenilin-1 that cause autosomal dominant early-onset AD [126]. To date, at least five lines that bear mutant human APP genes, that is, Tg2576, PDAPP, APP23, TgCRND8, and J20, have been reported and are widely used. These transgenic mouse lines differ in terms of genetic characteristics (e.g., different mutations, promoters, and/or genetic backgrounds), yielding different transgene expression levels and varying severity of AD-like pathology. Given its wide usage as a mouse model of AD-like pathology, we adopted Tg2576 mice originally developed by Karen Hsiao and colleagues in the mid-90s [127]. This mouse line overproduces human A*β*_1-40_ and A*β*_1-42_ and develops progressive *β*-amyloid deposits and learning and memory impairment beginning at 9-10 months of age [127–129]. To examine a possible role of S100B in the progression of AD-like pathology, we undertook a genetic approach to overproduce S100B by crossing transgenic mice expressing human S100B (TghuS100B mice) [86] with Tg2576 animals [127] to yield four genotypes of littermates: Tg2576, Tg2576-huS100B, TghuS100B, and wild-type. We then examined AD-like pathology in aged animals, including brain parenchymal and cerebral vascular *β*-amyloid deposits, A*β* levels, *β*-amyloid deposit-associated gliosis (astrocytosis and microgliosis), and pro-inflammatory cytokines.

We initially noted that the huS100B transgene exacerbated age-dependent cerebral amyloidosis, including brain parenchymal and cerebral vascular *β*-amyloid deposits in bitransgenic mice. We also undertook a biochemical approach to measure different forms of A*β* peptides. Consistent with histological results, we noted elevated soluble and insoluble A*β*_1-40_ and A*β*_1-42_ abundance. In brain areas examined early on during the course of A*β* deposition (at 9 months of age), we observed that Tg2576 mice had mainly dot-like *β*-amyloid deposits (<5 *μ*m in maximum diameter); by contrast, Tg2576-huS100B mice had large-sized *β*-amyloid plaques (>50 *μ*m in maximum diameter) that were already present at this early age. In addition, as we did not observe *β*-amyloid deposits in Tg2576 or Tg2576-huS100B mice at an even earlier age (7 months), the main difference between bigenic and singly-transgenic Tg2576 mice is higher plaque burden in the former, but not that amyloid deposition is initiated earlier in Tg2576-huS100B mice. Our findings can thus be interpreted as accelerated AD-like pathology by up to 4 months of age in TghuS100B-Tg2576 versus Tg2576 mice.

We also noted augmented astrocytosis and microgliosis, elevated levels of endogenous mouse S100 expression, and increased levels of pro-inflammatory cytokines (TNF-*α* and IL-1*β*) as early as 7–9 months of age in bigenic animals—prior to the onset of frank cerebral amyloid deposits in Tg2576 mice. Others have reported that gliosis is consequent upon progressive cerebral amyloid burden in AD model mice, given that glial activation is proportional to *β*-amyloid plaque load [130–132]. However, our finding that gliosis precedes increased *β*-amyloid plaque deposition in Tg2576-huS100B mice instead suggests that S100B-induced glial inflammatory responses drive accelerated *β*-amyloid load in these animals. This conclusion is strengthened by our findings that Tg2576-huS100B mice have significantly enhanced astrocytosis and microgliosis as compared to Tg2576 animals, both during initiation of cerebral amyloidosis (at 9 months of age) and at an age prior to *β*-amyloid deposition (at 7 months of age). However, it should be noted that we also measured pro-inflammatory cytokine mRNAs including TNF-*α*, IL-1*β*, IL-6, and mouse S100B at 7 months of age, and were not able to detect any consistent differences in these cytokines between the four groups of littermates. Thus, while the huS100B and Tg2576 transgenes synergize on increasing glial surface activation markers prior to formation of *β*-amyloid plaques (i.e., at 7 months of age), these two transgenes do not seem to cooperatively affect expression of pro-inflammatory cytokines until 9 months of age in this model.

**Figure 4:**
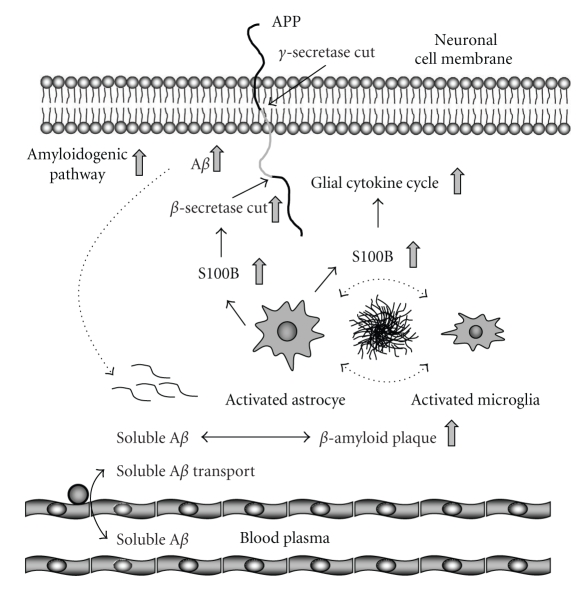
Diagrammatic representation of brain amyloid-*β* (A*β*) production/clearance, the harmful actions of S100B on A*β* production, and resultant effects on brain and plasma A*β* levels. In the amyloidogenic pathway, A*β* is primarily produced in neurons from sequential endoproteolytic cleavage of the type 1 transmembrane glycoprotein, *β*-amyloid precursor protein (APP), by *β*- and *γ*-secretases. Subsequently, soluble A*β* is secreted into the extracellular space, and then enters into a dynamic equilibrium between soluble and deposited (insoluble, *β*-amyloid plaque) forms. Continual transport of soluble A*β* occurs into and from the plasma. S100B enhancement of *β*-secretase activity promotes A*β* production, resulting in higher soluble and deposited A*β* in the brain. In addition, S100B promotes a damaging glial cytokine cycle through activation of astrocytes and microglia, resulting in enhancement of the amyloidogenic pathway.

The role of inflammatory responses and glial activation in the AD pathological process is multifarious, and studies have shown both beneficial and harmful effects of glial activation on AD-like pathology in mouse models depending on the stimulus [90, 114–117, 122, 132]. These results have led to the conclusion that there are various forms of gliosis in the context of AD, and not all forms of glial activation are damaging—some are likely even therapeutically advantageous [26, 123, 133]. The lines of evidence presented above suggest that brain pro-inflammatory events associated with S100B-dependent glial activation in close proximity to *β*-amyloid plaques lead to exacerbation of AD-like pathology, supporting the notion that chronic and prolonged activation of glia is detrimental in the context of neurodegenerative disease [81, 87, 134]. Such findings lend further support to the hypothesis that the astrocyte-derived protein, S100B, exacerbates the pro-inflammatory cytokine cycle, AD-like pathology, and associated brain injury [88–90]. A model depicting brain A*β* production/clearance, the harmful actions of S100B on A*β* production, and resultant effects on brain and plasma A*β* levels is shown in Figure 4.

Astrocyte-derived S100B promotes neurite extension and contributes to synaptogenesis and synapse remodeling in the developing and in the mature brain under physiological conditions [31, 32, 34, 38]. However, in the context of brain pathology such as AD, S100B may confer maladaptive neuritic changes in *β*-amyloid plaques. Supporting this notion, it has been reported that astrocytic overexpression of S100B drives dystrophic neurite outgrowth, culminating in the formation of neuritic plaques in Down's syndrome [135], and that S100B induction precedes appearance of neuritic *β*-amyloid plaques in the PSAPP AD mouse model [101]. Thus, S100B may drive conversion of diffuse, nonfibrillar *β*-amyloid deposits to neuritic *β*-amyloid plaques [30]. It is noteworthy that, while *β*-amyloid plaque morphometric analysis disclosed significant increases in small, medium, and large plaque size subsets in Tg2576-huS100B mice, we consistently noted the greatest exacerbation in medium- and large-sized plaques. Further, the huS100B transgene produced a number of “gigantic” *β*-amyloid plaques (>150 *μ*m) in aged bigenic mice. These data can be interpreted as consistent with a role for S100B in the maturation of *β*-amyloid deposits.

In addition to affecting cerebral parenchymal A*β* burden, the huS100B transgene also promotes cerebral vascular *β*-amyloid deposits. While A*β* synthesis has classically been regarded to predominantly take place at neuronal synapses, it has recently been reported that reactive astrocytes surrounding *β*-amyloid plaques express *β*-secretase in AD patients and in Tg2576 mice [136, 137]. Thus, the huS100B transgene may directly impact astrocytic amyloidogenic APP metabolism. This would explain both the exacerbation of parenchymal and cerebrovascular A*β* deposits in bigenic mice. But, does S100B accelerate cerebral amyloidosis by directly promoting amyloidogenic metabolism of APP? To evaluate this possibility, we examined amyloidogenic *β*-carboxyl terminal fragment (*β*-CTF; also known as C99) and amino-terminal APP cleavage products (soluble APP*β*) and noted increases in both species in Tg2576-huS100B versus Tg2576 mice. We also found increased levels of *β*-site APP cleaving enzyme 1 (BACE1; commonly known as *β*-secretase), and enhanced enzymatic activity in brain homogenates from Tg2576-huS100B mice. Thus, in addition to its ability to promote brain inflammatory responses, our data show that S100B has a previously unappreciated role in directly promoting amyloidogenic APP processing.

The results discussed above using an *in vivo* genetic approach can be interpreted as definitive evidence that forced expression of human S100B exacerbates AD-like pathology, including brain parenchymal and cerebral vascular *β*-amyloid deposits, A*β* levels, *β*-amyloid deposit-associated astrocytosis and microgliosis, and pro-inflammatory cytokines [138]. These data suggest that inhibition of S100B represents a novel therapeutic target for AD. To further explore this tantalizing possibility, we took a pharmacological approach to blocking S100B biosynthesis, as detailed below.

### 4.2. Arundic Acid Ameliorates Cerebral Amyloidosis and Gliosis in Alzheimer Transgenic Mice

We have previously shown that arundic acid negatively regulates S100B synthesis by suppressing mRNA expression in activated astrocytes [93]. In addition, arundic acid exerts further effects both *in vitro* and *in vivo* on other biomolecules as described above. To examine whether inhibiting reactive astrocyte-derived S100B might impact progression of AD-like pathology in the Tg2576 AD mouse model [127], we orally administered the S100B biosynthesis blocker, arundic acid, to Tg2576 mice for 6 months, commencing at 12 months of age (when *β*-amyloid plaques are initially present in this AD mouse model). Strikingly, arundic acid significantly suppressed cerebral amyloidosis in treated Tg2576 mice. Importantly, *β*-amyloid deposits were significantly decreased in arundic acid-treated Tg2576 mice, irrespective of size. Concurrent reductions in brain levels of both soluble and insoluble A*β* species corroborated our histopathological observations. Notably, *β*-amyloid plaque-associated reactive astrocytosis and microgliosis were also significantly inhibited, suggesting that arundic acid treatment arrested the feed-forward pro-inflammatory cytokine cycle that would be expected to enable astrocytic activation of microglia.

In terms of a direct mechanism for the beneficial effects of arundic acid on reducing AD-like pathology, a number of possibilities deserve consideration. Firstly, the drug might alter APP expression in Tg2576 mice, either by affecting endogenous murine APP levels or by modulating the hamster prion promoter-driven mutant human APP transgene [127]. Yet, APP expression in brain homogenates was comparable between arundic acid-treated and vehicle-treated Tg2576 mice, making this explanation seem unlikely. Secondly, as long-term administration of indomethacin, a key inhibitor of the inflammatory mediator NF-*κ*B, significantly reduces cerebral amyloidosis in Tg2576 mice [139], and NF-*κ*B is downstream of S100B [58], it is possible that arundic acid-induced inhibition of NF-*κ*B activity may mitigate amyloidosis in Tg2576 mice. In fact, we previously observed that lipopolysaccharide-induced activation of NF-*κ*B in cultured astrocytes was significantly attenuated by arundic acid [93]. Thus, it seems likely that NF-*κ*B inhibition is at least partly responsible for arundic acid-induced reduction of A*β* pathology in this scenario. Thirdly, biochemical evidence of decreased brain levels of soluble and insoluble A*β*—against a backdrop of unaltered APP production—suggests inhibition of amyloidogenic APP metabolism in arundic acid-treated Tg2576 mice. Whether this effect occurs via direct inhibition of *β*-secretase, or via an indirect mechanism, remains to be elucidated. Because, as mentioned above, astrocytes express *β*-secretase and have been directly implicated in amyloidogenic APP metabolism in the context of AD [136, 137], a direct mechanism of action remains possible.

Lastly, in addition to the pro-inflammatory effects of astrocytic S100B, its effects on neural reparative processes might be related to *β*-amyloid plaque development. It has been proposed that synaptic dysfunction occurs in both the prodromal and the clinical phases of AD [98], and excessive neuroplastic burden has been postulated to be a prime mover in the disease process [140]. It has been reported that astrocytic filopodia in tripartite synapses sense alterations in synaptic transmission, leading to their activation [141]. Activated astrocytes contribute to both reparative and destructive actions, which are considered to be at least partially mediated by altered levels of S100B [142]. Thus, pharmacological suppression of astrocytic S100B biosynthesis by arundic acid may have dual roles in delaying disease progression: in brain regions with relatively low levels of S100B, arundic acid might inhibit neurite extension in response to cerebral amyloidosis, and thereby decrease neuroplastic burden. By contrast, in brain regions where S100B is highly expressed, the agent may suppress the pro-inflammatory autotoxic loop [24, 30] and thereby reduce *β*-amyloid plaque maturation.

## 5. Concluding Remarks

In this paper, we have considered beneficial physiological functions and detrimental roles of the astrocyte-derived protein S100B in cerebral ischemia and in AD. Importantly, we propose that both of these neurological diseases share a common pathogenic mechanism—maladaptive astrocytic activation—where S100B acts as a perpetrator of neuroinflammation and neurotoxicity. Using both genetic and pharmacological approaches, we have produced evidence supporting the idea that enhanced and prolonged activation of astrocytes plays a detrimental role in the pathogenesis of cerebral ischemia and AD, and that astroctye-derived S100B is at the epicenter of these damaging cellular responses. In agreement with these lines of evidence, another group has demonstrated that S100B transgenic and knockout mice show worsening and attenuation, respectively, of ischemic brain damage [143]. In terms of AD, there is evidence that S100B transgenic mice show enhanced susceptibility to neuroinflammation and neuronal dysfunction induced by intracerebroventricular infusion of human *β*-amlyoid [144]. These and other studies unequivocally suggest that inhibiting astrocytic activation by pharmacological blockade of S100B biosynthesis may be a valuable therapeutic strategy to combat ischemic stroke and to delay onset and/or progression of AD. As with any pharmacological approach, it is important to note that there are advantages and disadvantages to modulating astrocytic activation via targeting S100B. Since available data are limited using genetic approach(es) to elucidate the possible role(s) of S100B in CNS health and disease, further studies using S100B and RAGE knockout mice, either singly or doubly-deficient for these genes, are warranted. The availability of selective S100B biosynthesis inhibitors such as arundic acid is expected to further translational research for neurological and neurodegenerative diseases, and perhaps other disorders, such as inflammatory bowl disease, which are S100B overexpresssion-related. Such future studies will go on to address the tantalizing possibility of next-generation therapeutics for ischemic stroke and AD aimed at the pleiotropic S100B pathway.
